# Tandemly Arrayed Genes in Vertebrate Genomes

**DOI:** 10.1155/2008/545269

**Published:** 2008-09-21

**Authors:** Deng Pan, Liqing Zhang

**Affiliations:** ^1^Department of Computer Science, Virginia Tech, 2050 Torgerson Hall, Blacksburg, VA 24061-0106, USA; ^2^Program in Genetics, Bioinformatics, and Computational Biology, Virginia Tech, 2050 Torgerson Hall, Blacksburg, VA 24061-0106, USA

## Abstract

Tandemly arrayed genes (TAGs) are duplicated genes that are linked as neighbors on a chromosome, many of which have important physiological and biochemical functions. Here we performed a survey of these genes in 11 available vertebrate genomes. TAGs account for an average of about 14% of all genes in these vertebrate genomes, and about 25% of all duplications. The majority of TAGs (72–94%) have parallel transcription orientation (i.e., they are encoded on the same strand) in contrast to the genome, which has about 50% of its genes in parallel transcription orientation. The majority of tandem arrays have only two members. In all species, the proportion of genes that belong to TAGs tends to be higher in large gene families than in small ones; together with our recent finding that tandem duplication played a more important role than retroposition in large families, this fact suggests that among all types of duplication mechanisms, tandem duplication is the predominant mechanism of duplication, especially in large families. Finally, several species have a higher proportion of large tandem arrays that are species-specific than random expectation.

## 1. Introduction

Although the importance of duplicated genes in providing raw materials for genetic innovation has been recognized since the 1930s and is highlighted in Ohno's book *Evolution by Gene Duplication* [1], it is only recently that the availability of numerous genomic sequences has made it possible to quantitatively estimate how many genes in a genome are generated by gene duplication. For instance, it has been estimated that about 38% of the genes in the human genome and 49% of the *Caenorhabditis elegans* genome arose from gene duplication [2, 3]. It is almost certain that current estimates of the extent of gene duplications are low, as many duplicated genes may have diverged to such a great extent that their common origin can no longer be recognized.

Known mechanisms of gene duplication include unequal crossover (or equivalently, tandem duplication), retroposition, and segmental (or genome) duplication [4]. Unequal crossover consists of chromosomal mispairing followed by the exchange of DNA between nonhomologous regions and resulting in either gene duplication or gene deletion [5]. Retroposition refers to reverse transcription of the mRNA transcript of a gene into double-stranded DNA followed by insertion of the double-stranded DNA into a location typically distant from the original gene. Genome duplication in vertebrates is not as frequent as that in plants. According to the two-round genome duplication hypothesis, the last possible genome duplication in vertebrates occurred more than 400 million years ago [6]. Recent segmental duplications cover only about 2% of the mouse genome [7] and 4% of the human genome [8] and usually do not contain genes [9]. Recently, some general studies of gene duplications have been undertaken (e.g., [10]), as well as specific computational identification and characterization of retrotransposed duplicated genes with respect to their location and dynamics in species such as human and mouse [11, 12]. There have also been studies of duplicated genes generated through unequal crossover (tandem duplication) in *C. elegans* [13], *Arabidopsis thaliana* [14], *Oryza sativa* [15], and several mammals [16].

Our current study focuses on tandemly arrayed genes (TAGs) in available vertebrate genomes. Tandem duplication has been shown to act as the driving evolutionary force in the origin and maintenance of gene families [17] and has been a common mechanism of genetic adaptation to environmental challenges in organisms such as bacteria [18], mosquitoes [19], plants [20], and mammals [21]. TAGs constitute a large component of several eukaryotic genomes. For example, at least 10% of the genes in the genomes of *C. elegans*, *Arabidopsis thaliana*, and human are TAGs [13, 14, 16]. TAGs can either promote genomic diversity to enhance disease resistance, satisfy the requirement for a large amount of a gene product, or contribute to the fine-tuning of developmental stages and physiological functions [1, 22].

In this work, we performed a genome-wide survey of the TAGs in 11 completed or nearly completed vertebrate genomes. We provided some general statistics regarding the number of genes in these genomes that belong to TAGs, the contribution of TAGs to the total duplications, TAG size (i.e., how many genes are in an array) distributions, gene transcription orientations in TAGs, and the contribution of tandem duplication in the make-up of gene families of different sizes. We also identified species-specific TAGs and compared their distribution among species.

## 2. Results

**Figure 1:**
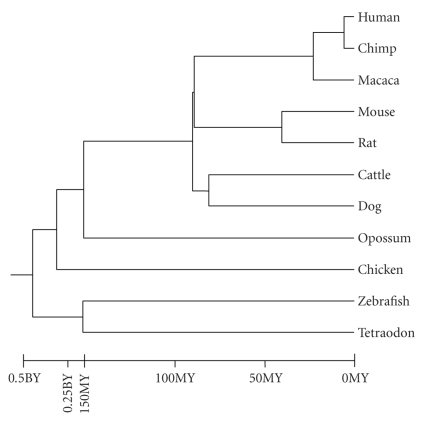
Phylogeny of the eleven species in this study.

The summary statistics are presented in Table 1. There are a total of 285 801 putative genes in the 11 genomes. Figure 1 (adapted mostly from [23]; the divergence time between zebrafish and tetraodon is from [24]) shows the phylogeny of these species. On average, each genome has 25 982 genes, with human (31 185) and chicken (19 399) having the most and least number of genes, respectively. The number of genes that have been assigned to a specific chromosome location in the 11 species reduces to 255 293. More than 90% of the genes have been assigned to a known location in the genome assembly for human, chimp, mouse, rat, macaca, dog, opossum, and zebrafish, whereas only 69%, 82%, and 55% have been assigned for cattle, chicken, and tetraodon, respectively. The numbers of gene families range from 2297 (chicken) to 4127 (zebrafish), and the numbers of genes contained in these families range from 7199 (chicken) to 20 187 (zebrafish) among all species. Thus, gene families on average contain about 3 to 5 members.

### 2.1. Spacers in TAGs

**Figure 2:**
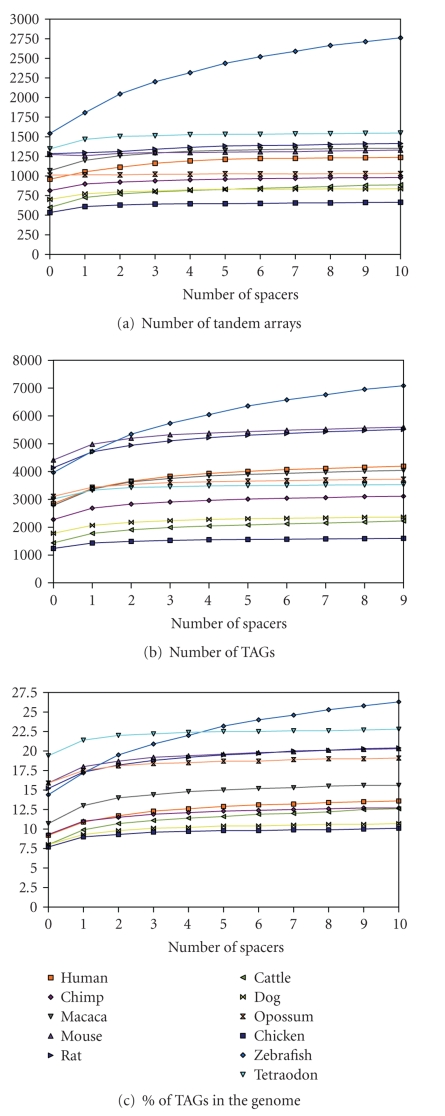
Distribution of tandem arrays and TAGs as a function of maximum number of spacers allowed.

TAGs are usually defined as genes that are duplicated tandemly on chromosomes. Spacers are genes that are not homologous to the members of TAGs (see Section 5 for details). Allowing different numbers of spacers between two members of an array will result in different numbers of TAGs. Figure 2 shows TAG statistics with respect to different numbers of spacers. There are three general patterns. First, for all species, the number of tandem arrays increases with the number of spacers allowed in the array, although the extent of increase varies among species (Figure 2(a)). The zebrafish shows the highest extent of increase in the number of arrays with increase of the number of spacers (*P* < .01 for all of the one-tailed *t*-tests between zebrafish and other species). Similarly, the number of genes included in the tandem arrays also increases when more spacers are allowed in the TAGs (Figure 2(b)). Second, for most species, both the number of tandem arrays and the number of genes in the arrays show the sharpest increase when going from spacer 0 to 1, consistent with studies in *Arabidopsis thaliana* and rice [14, 25]. Third, the similarities in these two quantities (the number of tandem arrays and the number of genes in the arrays) among species reflect to certain extent their evolutionary distances (Figure 1). For instance, mouse and rat show a very similar pattern in both the number of arrays and the number of genes; so do human and macaca, and dog and cattle. The zebrafish appears to be the most distinct of the remaining species, having the highest numbers in both arrays and genes for almost all TAG definitions (*P* < .01 for all pairwise *t*-test between zebrafish and the other species), perhaps because the zebrafish has undergone recent genome duplications so that the number of tandemly arrayed genes are much larger than for other species. An exception is seen in the chimp where, despite its being the most closely related to the human, there is a much greater divergence in the two quantities from human than in macaca from human. The quality of the chimp genome assembly has been known to be poor, which might explain the strange pattern that we observe here.

The percentages of TAG genes range from about 8% to 19% among all species when no spacers are allowed in TAGs, from about 10% to 26% when allowing 10 spacers. Therefore, TAGs contribute to a large proportion of genes in vertebrate genomes (Figure 2(c)). As previous and current genome-wide studies of TAGs suggest that allowing 1 spacer between array members is a good compromise between stringency and gene coverage, we report for the rest of the study only the results on TAGs that have at most 1 spacer. Note that according to our definition, allowing at most 1 spacer means that every pair of the neighboring genes in a TAG array has at most 1 spacer; therefore, the array can have more than 1 spacer in total.

### 2.2. Contribution to gene duplication

Tandem duplication has been commonly cited in the literature as one of three major mechanisms of gene duplication [4]. However, a quantitative evaluation of its contribution to duplications in the vertebrate genomes has not been available until our recent report [16]. Here, we also examined the percentage of duplicated genes that are in tandem arrays in these 11 genomes. Results are shown in Table 1. TAGs not only make up nearly 20% (9%–21%) of the genes, but also account for up to one third (18%–34%) of all duplications in these genomes.

### 2.3. Size of tandem array

Table 2 shows the distribution of tandem array sizes (i.e., the number of genes in a tandem array) and the percentages of TAG genes in each size category. Among all species, about 60% to 83% of the tandem arrays are of size two, that is, having only 2 members in the arrays. The proportions of tandem arrays of larger sizes decrease rapidly after size two. Mouse (30%), rat (34%), and opossum (38%) have the least proportions of two-member arrays, in contrast to 41%–73% for all remaining species. Mouse, rat, and opossum tend to have more larger arrays. In fact, the average number of genes per array ranges from 3.4 to 4.0 in mouse, rat, and opossum, from 2.4 and 3.2 in the remaining species.

### 2.4. TAG orientations

Table 3 shows the statistics of three types of gene transcription orientations (parallel →→ or ←←, convergent →←, and divergent ←→) for both genomes and TAGs. The proportions of neighboring genes with three different transcription orientations in the genome are very similar among all species, with parallel transcription orientation being the major type (varying within a narrow range of about 50%–57%) and equal proportions of convergent and divergent transcription orientations (both about 22%–25%). In contrast, for all species, the majority of gene pairs in TAGs have a parallel transcription orientation, ranging from about 72% to 94%, much higher than those in the genomes (*P* < .01, *t*-test). The proportions of convergent and divergent transcription orientations in TAGs are similar, ranging from 3% to 14% among species. Statistical tests show that the distribution of the three types of transcription orientations in TAGs is significantly different from that of all genes in the genome (the chi-square Goodness-of-Fit test: *P*-value < 1E-36 for all species).

### 2.5. TAGs in gene families

**Figure 3:**
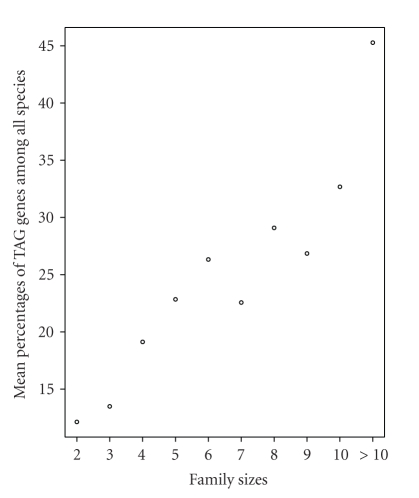
Contribution of TAGs to families of different sizes.

Table 4 shows the proportions of duplicated genes that belong to TAGs in gene families of different sizes. There is a clear trend that, as family size gets larger, the proportion of TAGs in the families also increases. For instance, in gene families of size two (i.e., families that have two members), only around 10% of gene members belong to TAGs (except for tetraodon), whereas in families of sizes > 10, 35%–60% of the members belong to TAGs. Figure 3 shows the relationship between family sizes and mean percentages of TAGs (averaged over all species). Tests of correlation show that the percentages of TAGs in gene families are positively correlated with gene family sizes (Pearson's correlation coefficient *r* varies from .78 to .94 among species, all *P*-values <.008). The correlation remains significant even after removing the family size >10 that includes all families with >10 genes for all species (all *P*-values <.05).

We also examined the homologous tandem arrays (the TAGs that belong to the same Ensembl gene family) across all the species. Due to the complex homologous relationship between the members of TAGs within and across species (see Section 3), we did not perform the standard phylogenetic analysis. Instead, to explore the relationship between TAGs across species, we clustered the species based on distribution of the number of TAGs in the same families across these species by the K-means clustering method [26]. Specifically, each row of the input matrix for K-means clustering contains a vector with the numbers in the vector corresponding to the number of TAG occurrence in each of the gene families in a particular species. Therefore, we used the K-means clustering to take account of the information of all families in order to group the species that show similar TAG profiles. Our purpose is to test whether the clustering based on TAGs is congruent with the species tree (Figure 1). We set the number of clusters *K* from 2 to 4. When *K* = 2, the resulting two groups are {human, chimp, macaca, mouse, rat, opossum} and {cattle, dog, chicken, zebrafish, tetraodon}; when *K* = 3, the resulting three groups are {human, chimp, macaca, opossum}, {mouse, rat}, and {cattle, dog, chicken, zebrafish, tetraodon}; and when *K* = 4, the resulting four groups are {human, chimp, macaca, opossum}, {mouse, rat}, {cattle, dog, chicken, tetraodon}, and {zebrafish}. Compared with the species tree, it turns out that primate species (human, chimp, macaca) and murine species (mouse, rat) are always clustered correctly, but cattle and dog are more likely to be clustered with nonmammals.

### 2.6. Species specific tandem arrays

Studies have shown that species-specific duplications can play an important role in species-specific traits or life styles that enable species to adapt to certain environments (e.g., [27–29]). We studied species-specific tandem arrays (SSTAs), which are defined as the tandem arrays that are present in only one species while there may be no homologues or homologues are not tandemly arrayed in all the other species. Table 5 shows the summary of SSTAs in all species. There are about 10% SSTAs in mammals and more than 20% in nonmammals. The higher proportion of SSTAs in nonmammals may be mainly due to their much higher divergence from the most recent common ancestor in the species tree (Figure 1). We also used Gene Ontology (GO) annotations to see whether there are any GO categories that are highly enriched in the SSTAs. We found no apparent preference of specific GO functions even between closely related species, such as mouse and rat. But as not all SSTA genes have GO information, further evaluation is needed.

As SSTAs are more likely to be recently born than are the non-SSTA arrays that are shared by multiple species, we expect that under neutral evolution, the sizes of SSTAs (i.e., the number of genes in an array) should be on average smaller than the sizes of non-SSTA arrays. As most of the SSTAs are of size-two (Table 5), we expect that the proportion of SSTAs that are of size two should be higher than the proportion of non-SSTA arrays that are of size two. Only in chimp, macaca, rat, and dog is the proportion of size-two SSTAs significantly higher than that of size-two non-SSTAs, which means that the sizes of SSTAs in most of the species are not significantly smaller than the sizes of non-SSTA arrays.

## 3. Discussion

### 3.1. Contribution of tandem duplication

Here we performed a genome-wide survey of TAGs in 11 assembled vertebrate genomes. In summary, when using a stringent criterion for TAG identification (e.g., allowing at the most 1 spacer between array members), we observed a consistent pattern of tandem duplication contributing to the number of genes in the genomes and to genome wide duplications: on average, about 14% of the genes in vertebrate genomes are TAGs, and about 25% of all duplicated genes are tandemly arrayed.

These numbers most likely underestimate the extent of tandem duplication in these genomes. Our recent study shows that more than 25% to 40% of the recent gene duplications are generated by tandem duplications in human and mouse [30]. Therefore, it is likely that many old tandem arrays became invisible during evolution owing to various genome rearrangements. Meanwhile, one may wonder whether duplicated genes arising from duplication mechanisms other than tandem duplication could get scrambled during evolution and happen to be arranged as TAGs. However, as shown in our previous study, this possibility should have minimal effect on the TAG statistics because the probability that duplicated genes appear as TAGs by chance is very low, about 1 to 2 orders of magnitude lower than the actual extent of tandem duplication [16].

### 3.2. TAG transcription orientation

It has been shown that ∼80% and ∼88% of tandem arrays are in parallel transcription orientation in *Arabidopsis thaliana* and rice, respectively [25]. How this compares to the genome patterns in these species has not yet been studied. The vertebrate genomes show amazingly consistent patterns in the proportion of gene pairs in parallel, convergent, and divergent transcription orientations with a ratio of ∼2 : 1 : 1. In contrast, TAGs have much higher proportions of parallel orientation, ranging from about 72% to as high as 94%. Therefore, in both plants and animals, parallel orientation is the dominant type of transcription orientation in TAGs.

So why is there disproportionately less convergent and divergent transcription orientation in TAGs than in the genome? One explanation is that tandem duplications occur at a higher rate on the same strand than on different strands. Little is known about what determines the rates of tandem duplication on the same strand or different strands. Therefore, how much differential rates of tandem duplication between same-strand and different-strands contribute to the observed dominance of parallel orientation across all the studied species remains an open question. Another possible explanation is related to long inverted repeats (LIRs). It has been shown that LIRs can substantially increase genome instability. For example, in the mouse, LIRs in germ lines can lead to elevated genome rearrangements due to increased levels of illegitimate recombination, gene conversion, and deletion mediated by LIRs [31, 32]. In the human, several genetic diseases have been reported to be caused by illegitimate recombination and deletion induced by LIRs [33]. In the case of TAGs, tandem duplicated genes on opposite strands (in convergent or divergent orientation) are essentially LIRs, and their initial high sequence identity might increase the level of illegitimate recombination and various genome rearrangements. The increased genome instability might have a disastrous effect on the individuals that carry tandem duplication; strong negative selection against the duplication will reduce the fixation probability of tandem duplication in the population. This may at least in part explain why we observe a much lower proportion of TAGs with convergent and divergent orientations than in the genome. Meanwhile, the fact that there are still some TAGs with convergent or divergent orientation can also be explained by LIR-mediated changes. It has been observed that illegitimate recombination events induced by LIRs sometimes result in asymmetric deletion that eliminates the central symmetry of LIRs [31, 32]. When the deletion does not have a negative effect on the function of the genes, for example, when the deletion happens to be located in introns, the elimination of the symmetry in the LIRs can actually prohibit further illegitimate rearrangements and reduce the levels of gene conversion. Consequently, the LIRs, that is, tandem duplicated genes on opposite strands, no longer pose a threat to genome stability and thus can be fixed in the population [5, 31]. More research needs to be done to determine the causes of the higher proportion of TAGs in parallel orientation than the genome average.

### 3.3. Tandem array sizes

All plant and animal genomes that we have studied so far show that the majority of tandem arrays contain only two members. It is likely that large arrays are destroyed by various genome rearrangements and become smaller arrays over time, which might be the case for most of the tandem arrays. For the large TAG arrays such as the 18S and 28S ribosomal RNA genes in the vertebrates [22], mechanisms such as continued concerted evolution (including unequal crossover and gene conversion) and natural selection need to act on the arrays to prevent array-size decay.

The fluctuation of array sizes has been observed in natural populations of many species such as humans [34] and flies [35]. Empirical evidence also suggests that the fluctuation can produce visible phenotypic effects and sometimes can be detrimental. For example, in *Drosophila melanogaster*, 18S and 28S rRNA genes contain 150 to 250 tandemly arranged repeats in wild-type flies [35, 36] and individuals carrying a lower copy number than the wild-type have so-called bobbed mutations, characterized phenotypically by having small bristles, abdominal etching, and developmental delay [37, 38]. These studies show that the size of tandem arrays is important in the normal function of organisms and the fluctuation of array sizes might be selected against.

At the same time, a variety of mechanisms can reduce or prevent size change in a tandem array. For example, insertion of irrelevant genes (i.e., genes with no homology to the array members) into the array may effectively reduce the frequency of unequal crossovers. The divergence of array members can also reduce the frequency of unequal crossover. Therefore, observation on array sizes across multiple animal and plant genomes reflects not only a snapshot of current genomes, but also most likely a stable state of TAGs as a result of joint processes of selection, drift, and mutation on the arrays.

### 3.4. Tandem duplication and family size

The positive correlation between the extent of tandem duplication and the sizes of gene families (Figure 3 and Table 3) indicates that the contribution of TAGs to gene families of different sizes weighs more in large gene families than smaller ones. It may also be possible that large gene families can have higher percentage of genes belonging to TAGs than small gene families simply by chance. We performed a permutation test to see whether chance alone can produce such a strong association between family sizes and TAG percentages. We simulated 10 000 pseudogenomes, each of which has the same number of gene families and distribution of family sizes as the studied genomes. We randomly assigned the chromosome location of all the genes in the pseudogenomes and determined the percentage of TAGs for all the family sizes. We then examined the correlation between the percentage of TAGs and family sizes and found that indeed they are correlated. However, the percentage of TAGs in the simulation is much lower than our observation in all sizes. For example, in human, we observed about 34% of TAGs in the gene families of size 10, while only about 4% in our simulated distribution. In fact, the simulated percentages of TAGs in all the gene family sizes are about 10 times lower than the actual observations. Thus, it is clear that even though chance does contribute to the positive correlation between the extent of tandem duplication and the sizes of gene families, it is not a determining factor.

Consistent with the current observation, our recent study shows that tandem duplication generated more duplicated genes than retroposition did in large families in both humans and mice [30]. Many genes in large families such as olfactory receptor genes and zinc finger protein genes are generated by tandem duplication. The question is why tandem duplication has played a more important role in large families than in small families.

To answer the question, we need to compare the differences among various mechanisms of gene duplication. There are three major mechanisms of gene duplication: genome duplication, retroposition, and tandem duplication [4]. Among the three, genome duplication happens the least frequently in animals. Moreover, it doubles the copy number of all genes and thus should have a similar contribution to different-sized families. In contrast, tandem duplication is more frequent and more specific as it duplicates only specific genes instead of every gene in the genome. It may be difficult for a gene to change from single copy to duplicated states because sequence homology around the gene, required by unequal crossover to generate tandem duplication, is not always present. However, once a tandem duplication occurs, it is easy for unequal crossover to quickly expand the array due to the availability of sequence homology. Thus, tandem duplication has the advantage of being fast and easy in generating a large number of genes and providing opportunities for the divergence and refinement of gene function among duplicated members.

It has been shown that retroposition seems to be more active in highly expressed genes in germline cells [39, 40]. However, members in large gene families are not necessarily highly expressed. Our recent study suggests that the expression level seems to be more important than gene family size in determining what genes get retroposed [41]. Moreover, due to the nature of retroposition, the retroposed copy does not have ancestral regulatory regions and its survival is thus dependant upon the probability of being able to capture a regulatory region. The large amount of retroposed pseudogenes in the human and mouse genomes suggests that the probability of survival of the retroposed copy is very small. In contrast, many fewer pseudogenes are generated by tandem duplication [42], suggesting that the survival rate of tandem duplication is much higher than the retroposed genes. Therefore, as unequal crossover is the most efficient mechanism to generate and maintain gene copies among the three major gene duplication mechanisms, it may explain why TAGs are more frequent in large families than in small ones.

### 3.5. TAG homology and SSTAs

Identifying the homologous relationships (orthologous and paralogous) for TAGs across species is a challenging task. Frequent gene conversion within tandem arrays [43] and gene losses and gains of different array members in different species make genome-wide orthologue assignments computationally intractable [44]. One good example that shows the difficulty of homology assignments in TAGs can be seen in the HOX genes. Numerous studies of these genes have shown that there is a tremendous amount of variation in the number of HOX clusters in different species. Moreover, there are losses and gains of different members in different species and frequent gene conversion or concerted evolution in some members (e.g., [45–48]). Computationally, it is nearly impossible to identify a correct homology relationship for these genes across multiple species. There have been computational attempts to infer an evolutionary history of tandem repeated sequences in multiple species (e.g., [49–51]). However, it is clear that correct inference of evolutionary history relies on correct homology assignment, which remains a computationally challenging problem.

To circumvent the homology assignment problem, we studied two aspects regarding the evolution of TAGs in the 11 species that do not require identification of exact homology relationships among TAGs. The first aspect is to examine the evolutionary closeness of the 11 species in terms of TAG quantities in different gene families. There are two TAG quantities that one can describe for a particular gene family. One is the total number of arrays in the family and the other is the total number of tandemly-arrayed genes in the family. It is expected that the two quantitative descriptions should show similar evolutionary closeness to what the species tree reflects. Our K-means clustering result suggests that the two quantities, to a large extent, are able to reflect the phylogenetic relationship of the species. The exception is the grouping of dog and cattle, which is always clustered with the nonmammals. A possible explanation is that many genes have not yet been annotated in these two species, especially those mammalian-specific TAGs, which makes them appear closer to nonmammals. Alternatively, it may also mean that some ancestral mammalian TAGs are broken up in dog and cattle.

The second aspect is related to species-specific tandem arrays (SSTAs). Apparently, the definition of SSTAs determines that SSTA statistics are sensitive to the number, the kind, and the annotation quality of the species that are sampled. For example, it is expected that the more species included in a sample, the less likely an array will be an SSTA. Meanwhile, the number of SSTAs in a certain species is directly influenced by the species' distance to its closest related species in the sample. For instance, the number of SSTAs in human in the human-mouse-rat sample will certainly be higher than the number of SSTAs in human in the sample that also includes chimp. Moreover, if the annotation qualities of the two species are different, for instance in the case of human and chimp, there would be more SSTAs in the better annotated species human than in the less well-annotated species chimp.

Despite these caveats, study of SSTAs, or more generally, species-specific duplication, can potentially provide insight into the adaptive evolution of species-specific traits and life styles. For example, one of the human SSTAs, the sperm protein associated with the nucleus on the X chromosome *SPANX* gene family, containing two tandem arrays with a total of 6 genes, has been reported to have gone through rapid evolution and amplification in hominids [52]. Our analyses show that the proportion of tandem arrays with more than two members in SSTAs is significantly higher than that in non-SSTAs in some of the studied mammals, which suggests that nonneutral forces may maintain relatively recent-born arrays. However, caution must be taken to interpret the results as the species sampling is not homogeneous and some of the SSTAs might not be truly species-specific due to the deep divergence.

## 4. Conclusions

We have provided a quantitative account of TAGs and their contribution to duplications in vertebrate genomes. This is a first step towards understanding the evolution of these genes. As it has been increasingly realized that how genes are arranged on chromosomes plays an important role in determining gene function, TAGs stand out for their unusual spatial arrangement. Future research can be directed towards further understanding the intricate differences of tandem duplication from other types of duplication and the impact on the ultimate fate of duplicated genes.

## 5. Materials and Methods

There are altogether 11 vertebrate genomes assembled and available in Ensembl Version 41 (http://ensembl.org/). Therefore, we focused on these 11 species including human (*Homo sapiens*), chimp (*Pan troglodytes*), mouse (*Mus musculus*), rat (*Rattus Norvegicus*), macaca (*Macaca mulatta*), cattle (*Bos taurus*), dog (*Canis familiaris*), opossum (*Monodelphis domestica*), chicken (*Gallus gallus*), zebrafish (*Danio rerio*), and tetraodon (*Tetraodon nigroviridis*). Previously, we studied TAGs in the genomes of human, mouse, and rat [16]. However, as the annotation quality has been continually improved over time and this paper is intended to be a comprehensive overview of TAGs in all available vertebrate genomes, we reanalyzed these species using the latest version 41 as well.

Annotation of genes for all 11 species was obtained using Ensembl Biomart (http://ensembl.org/). The total number of genes is shown in Table 1. Genes annotated as unknown and mitochondrial were removed and only those with known chromosome location were kept, as we needed the information to determine TAGs. We also required that each gene should be equal to or longer than 300 nucleotides. Family information was also obtained using Ensembl Biomart. In Ensembl, gene families are clustered using Markov clustering algorithms (MCL) based on sequence similarities (see http://ensembl.org/ for details). All data were stored in MySQL database for subsequent analysis.

TAGs are usually defined as genes that are duplicated tandemly on chromosomes. Members of tandem arrays may be separated by other unrelated genes (called spacers). During evolution, various genome rearrangements, such as transposition and insertion of genes that are unrelated to array members (i.e., not through duplication), can disrupt the spatial arrangement of the TAGs. Allowing different numbers of spacers in between two members of an array will result in a different number of TAGs. For example, consider an array with the spatial arrangement of *A*_1_-*B*-*A*_2_-*A*_3_-*C*-*A*_4_-*A*_5_, where all *A*s are duplicated genes, and *B* and *C* are spacers. When allowing 0 spacers, we will have 2 tandem arrays with each having 2 members (*A*_2_ and *A*_3_; *A*_4_ and *A*_5_); allowing 1 spacer, we will have 1 array with 5 members (*A*_1_, *A*_2_, *A*_3_, *A*_4_, and *A*_5_).

To obtain TAGs, we sorted all the genes of each species chromosome by chromosome and indexed them in ascending order based on their physical locations. Let *d* denote the absolute difference of the indices between two genes on the same chromosome. *d* − 1 is equal to the number of spacers between these two genes *S*. When *S* = 0, it is a perfect TAG gene pair with no spacers. For certain *S*, we marked those gene pairs with *d* ≤ *S* + 1 and clustered them using a single-linkage algorithm, which ensures that within each tandem array, there exists at least one TAG link between any two array members. A TAG link is the relationship of two genes that can be seen as a TAG pair under the certain number of spacers allowed. We screened TAGs under each TAG definition (spacers 0–10) for every species.

## Figures and Tables

**Table 1:** Numbers of nuclear genes in genomes of species utilized.*TAGs are defined as having zero or one spacer gene.

Species	Genes	Annotated genes	Gene families	Annotated genes in families	TAGs*	%TAGs* in duplicated genes	%TAGs* in the genome
Human	31185	31126	3617	14473	3394	23.5%	10.9%
Chimp	25510	24522	3262	12376	2686	21.7%	11.0%
Macaca	27429	25990	3543	14439	3371	23.3%	13.0%
Mouse	27964	27736	3645	16091	4984	31.0%	18.0%
Rat	27233	27194	3510	16446	4712	28.7%	17.3%
Cattle	25977	17895	2616	9146	1779	19.5%	9.9%
Dog	22800	22257	3160	11480	2067	18.0%	9.3%
Opossum	21288	19598	3101	12195	3438	28.2%	17.5%
Chicken	19399	15966	2297	7199	1433	19.9%	9.0%
Zebrafish	28506	27457	4127	20187	4729	23.4%	17.2%
Tetraodon	28510	15552	2654	9702	3332	34.3%	21.4%

**Table 2:** The percentages of tandem arrays (PTA) and the corresponding percentages of TAGs (PTG) in each array size.

		Size of TAG									
Species	Statistics	2	3	4	5	6	7	8	9	10	>10
Human	PTA	65.4	13.6	6.6	4.4	3.4	1.0	1.0	1.0	0.6	2.8
	PTG	40.6	12.6	8.2	6.8	6.4	2.3	2.6	2.9	1.8	15.8

Chimp	PTA	65.3	16.9	6.6	3.2	3.0	1.1	0.9	0.7	0.8	1.6
	PTG	43.6	17.0	8.8	5.4	6.0	2.6	2.4	2.0	2.6	9.6

Macaca	PTA	70.0	15.2	5.6	3.2	1.3	1.3	0.8	0.5	0.4	1.7
	PTG	49.8	16.3	8.0	5.6	2.8	3.3	2.1	1.6	1.5	8.9

Mouse	PTA	59.7	14.2	6.9	3.7	3.3	2.5	1.6	1.8	1.0	5.2
	PTG	30.1	10.8	7.0	4.7	5.1	4.5	3.2	4.2	2.6	27.9

Rat	PTA	62.0	15.2	7.1	4.2	2.8	1.7	1.1	0.9	0.9	4.2
	PTG	34.1	12.5	7.8	5.7	4.6	3.3	2.4	2.3	2.5	24.7

Cattle	PTA	77.1	14.3	3.6	2.9	0.6	0.4	0.1	0.6	0.1	0.3
	PTG	63.0	17.5	5.8	5.9	1.3	1.2	0.4	2.0	0.6	2.2

Dog	PTA	77.2	13.0	3.5	1.0	1.3	1.3	0.4	0.6	0.3	1.4
	PTG	57.7	14.5	5.2	1.9	2.9	3.4	1.2	2.2	1.0	10.1

Opossum	PTA	64.6	15.1	6.0	3.4	2.8	1.6	1.1	0.5	0.3	4.6
	PTG	38.2	13.4	7.1	5.1	4.9	3.3	2.6	1.3	0.9	23.4

Chicken	PTA	81.8	11.3	3.4	1.5	0.8	0.5	0.2	0.0	0.3	0.2
	PTG	69.6	14.4	5.9	3.1	2.1	1.5	0.6	0.0	1.4	1.4

Zebrafish	PTA	78.0	11.0	5.0	1.8	1.4	0.6	0.4	0.3	0.2	1.3
	PTG	59.6	12.6	7.7	3.5	3.2	1.5	1.2	1.1	0.8	8.8

Tetraodon	PTA	82.8	12.2	3.1	1.0	0.2	0.1	0.1	0.3	0.0	0.1
	PTG	72.9	16.1	5.5	2.3	0.5	0.2	0.5	1.1	0.0	0.9

**Table 3:** Occurrence of parallel, convergent, and divergent orientations among gene pairs. Both absolute numbers and percentages are shown.

	Parallel		Convergent		Divergent	
	Genome	TAG	Genome	TAG	Genome	TAG
Human	15843	1379	7625	246	7634	266
	(50.9%)	(72.9%)	(24.5%)	(13.0%)	(24.5%)	(14.1%)

Chimp	12095	1062	6199	191	6203	209
	(49.4%)	(72.6%)	(25.3%)	(13.1%)	(25.3%)	(14.3%)

Macaca	13273	1252	6345	230	6351	238
	(51.1%)	(72.8%)	(24.4%)	(13.4%)	(24.5%)	(13.8%)

Mouse	14307	2401	6705	377	6703	365
	(51.6%)	(76.4%)	(24.2%)	(12.0%)	(24.2%)	(11.6%)

Rat	13967	2127	6604	353	6602	375
	(51.4%)	(74.5%)	(24.3%)	(12.4%)	(24.3%)	(13.1%)

Cattle	8833	605	4518	116	4514	117
	(49.4%)	(72.2%)	(25.3%)	(13.8%)	(25.3%)	(14.0%)

Dog	11116	802	5549	138	5553	141
	(50.0%)	(74.2%)	(25.0%)	(12.8%)	(25.0%)	(13.0%)

Opossum	9861	1641	4864	234	4864	225
	(50.3%)	(78.1%)	(24.8%)	(11.1%)	(24.8%)	(10.7%)

Chicken	8006	581	3963	60	3967	62
	(50.2%)	(82.6%)	(24.9%)	(8.5%)	(24.9%)	(8.8%)

Zebrafish	15228	1865	6102	261	6102	299
	(55.5%)	(76.9%)	(22.2%)	(10.8%)	(22.2%)	(12.3%)

Tetraodon	8806	1563	3363	52	3362	55
	(56.7%)	(93.6%)	(21.7%)	(3.1%)	(21.6%)	(3.3%)

**Table 4:** Percentages of TAGs in gene families of different sizes. Absolute numbers are omitted for clarity.

	Family size										
Species	2	3	4	5	6	7	8	9	10	>10	Correlation
Human	11.03	8.95	14.56	20.95	28.85	17.25	28.30	20.74	31.33	46.75	.84
Chimp	8.65	9.68	15.86	25.27	25.00	21.60	21.57	29.08	30.80	46.45	.90
Macaca	11.64	11.77	20.34	23.73	26.88	21.43	23.78	27.19	34.07	39.86	.92
Mouse	11.02	12.39	15.67	19.02	29.04	23.42	32.33	33.57	43.57	59.42	.94
Rat	10.36	13.05	19.42	23.71	24.52	19.23	35.58	19.22	36.67	48.46	.84
Cattle	9.60	12.60	16.95	23.82	28.30	19.12	28.06	32.03	22.50	37.19	.84
Dog	9.09	10.80	13.76	21.05	17.49	14.96	20.70	16.67	30.00	39.17	.84
Opossum	9.99	13.89	21.25	20.67	24.93	21.12	32.25	40.86	31.20	60.11	.88
Chicken	13.41	15.61	24.88	20.63	28.00	29.64	24.34	23.61	28.33	34.65	.82
Zebrafish	11.20	12.36	16.35	15.28	18.78	19.03	26.13	19.07	32.13	38.27	.90
Tetraodon	27.50	27.28	31.31	37.18	37.82	41.45	46.94	33.33	38.82	47.67	0.78

**Table 5:** Statistics of species-specific tandem arrays (SSTAs). The percentages in the parenthesis are shown as follows: ^a^the percentage of SSTAs to the total number of tandem arrays and ^b^the percentage of size-two SSTAs to the total number of SSTAs.

Species	Number of SSTAs^a^	Number of size-two SSTAs^b^	Hypergeometric test *P*-value
Human	75 (7.12%)	49 (65.33%)	4.62e-01
Chimp	14 (1.56%)	14 (100%)	0
Macaca	85 (7.08%)	78 (91.76%)	1.00e-07
Mouse	111 (8.83%)	63 (56.76%)	7.10e-01
Rat	92 (7.09%)	68 (73.91%)	4.48e-03
Cattle	79 (10.88%)	65 (82.28%)	9.49e-02
Dog	46 (5.96%)	45 (97.83%)	4.48e-06
Opossum	95 (9.36%)	66 (69.47%)	1.24e-01
Chicken	139 (22.79%)	118 (84.89%)	1.14e-01
Zebrafish	684 (37.85%)	366 (53.51%)	1
Tetraodon	584 (39.81%)	457 (78.25%)	1
